# Transcriptional Profiling Provides New Insights into Organ Culture-Induced Changes in Human Donor Corneas

**DOI:** 10.3390/ijms232314507

**Published:** 2022-11-22

**Authors:** Julian Wolf, Paola Kammrath Betancor, Philip Maier, Sonja Ute Heinzelmann, Jana Jiang, Clemens Lange, Thomas Reinhard, Günther Schlunck, Thabo Lapp

**Affiliations:** 1 Eye Center, Medical Center, Faculty of Medicine, University of Freiburg, 79106 Freiburg im Breisgau, Germany; 2 Omics Laboratory, Stanford University, Palo Alto, CA 94304, USA; 3 Department of Ophthalmology, Byers Eye Institute, Stanford University, Palo Alto, CA 94304, USA; 4 Ophtha-Lab, Department of Ophthalmology, St. Franziskus Hospital, 48145 Münster, Germany

**Keywords:** RNA sequencing, cornea, corneal transplantation, corneal graft, eye bank, organ culture, formalin-fixed paraffin-embedded

## Abstract

Corneal transplantation is one of the most common forms of tissue transplantation worldwide. Donor corneal tissue used in transplantation is provided by eye banks, which store the tissue in culture medium after procurement. To date, the effects of cell culture on human corneal tissue have not been fully elucidated. Using the 3′ RNA sequencing method for massive analysis of cDNA ends (MACE), we show that cultivation of corneal tissue leads to significant changes in a variety of molecular processes in human corneal tissue that go well beyond aspects of previously known culture effects. Functionally grouped network analysis revealed nine major groups of biological processes that were affected by corneal organ culture, among them keratinization, hypoxia, and angiogenesis, with genes from each group being affected by culture time. A cell type deconvolution analysis revealed significant modulations of the corneal immune cell profile in a time dependent manner. The results suggest that current culture conditions should be further refined and that prolonged cultivation may be detrimental. Recently, we showed that MACE enables transcriptional profiling of formalin-fixed and paraffin-embedded (FFPE) conjunctival tissue with high accuracy even after more than 10 years of storage. Here we demonstrate that MACE provides comparable results for native and FFPE corneal tissue, confirming that the technology is suitable for transcriptome analysis of a wide range of archived diseased corneal samples stored in histological archives. Finally, our data underscore the feasibility of bioinformatics cell-type enrichment analysis in bulk RNA-seq data to profile immune cell composition in fixed and archived corneal tissue samples, for which RNA-seq analysis of individual cells is often not possible.

## 1. Introduction

RNA sequencing-based transcriptome analyses provide an unbiased overview of the gene expression status in cells and tissues and thus their functional state. In recent years, the technology provided new insights into several healthy and diseased ocular tissues [1,2]. Recent single-cell RNA-seq analysis of healthy corneal specimens differentiated 21 corneal cell types [3]. However, less is known about transcriptional changes in human corneal diseases or in donor corneas stored in organ culture before transplantation. To this end, it is essential to clarify if formalin-fixed paraffin-embedded (FFPE) corneal tissue is suitable for bulk RNA sequencing, which would unlock the wealth of various diseased corneal FFPE samples available in histopathological archives and biobanks to transcriptomic analysis. In addition, FFPE-fixed healthy corneal specimens could then serve as readily available control tissue to investigate the effect of organ culture on unfixed human donor corneas. FFPE-induced RNA degradation occurs predominantly at the 5′ end of the RNA, a fact exploited by a specialized 3′-RNA-seq technology (massive analysis of c-DNA ends, MACE), which sequences only the 3′ end of the RNA. Furthermore, each RNA molecule is barcoded with a unique molecular identifier (UMI) prior to amplification, which reduces the PCR bias and allows accurate quantification of transcripts [4]. An earlier study suggested that MACE is feasible to determine the transcriptional profile of FFPE conjunctival tissue with high accuracy even after 13 years of storage [4]. However, it is still unclear whether MACE is also amenable to archived corneal samples, whose analysis is challenged by factors such as comparatively low cell and RNA quantities. We therefore bisected three human donor corneas, whereas one half was FFPE-processed and the other half remained unfixed. The transcriptional profile of the paired samples was subsequently analyzed using MACE, revealing no significant effect of FFPE processing on the transcriptional profile of human corneal samples. These results confirm that the technology is suitable for transcriptional profiling of a variety of diseased corneal specimens which are stored in histological archives and in addition allow us to apply FFPE-fixed healthy corneal specimens as control tissue to investigate the effect of organ culture on unfixed human donor corneas.

Corneal cultivation is routinely performed worldwide using human donor corneas prior to corneal transplantation [5,6,7]. Most of the evidence on cultivation effects available to date is focused on the effect of culture conditions on endothelial cell density (ECD) and corneal graft survival after transplantation. For example, grafts that were kept in culture for more than 21 days showed less subsequent rejection than grafts stored for shorter times [8]. This phenomenon is associated with the fact that the number of competent immune cells in the cornea decreases with time in culture [9]. Furthermore, it has been proposed to prepare tissue for transplantation by adding factors to the culture medium that affect the differentiation status of immune cells in the graft, thereby reducing the subsequent risk of rejection [9]. In animal experiments, successful approaches adding interleukin-10 (IL-10) to the corneal culture have been reported [10]. However, apart from endothelial and immune cells, it is still poorly understood which other molecular processes may be modulated by corneal tissue culture. Here, we investigate the cultivation-induced transcriptional changes in human donor corneas using MACE RNA sequencing, providing an unsupervised and high-resolution molecular profile, substantially exceeding the aspects of culture effects studied so far.

## 2. Results

### 2.1. Formalin Fixation Does Not Have a Significant Impact on the Transcriptional Profile of Human Corneal Tissue

**Figure 1:**
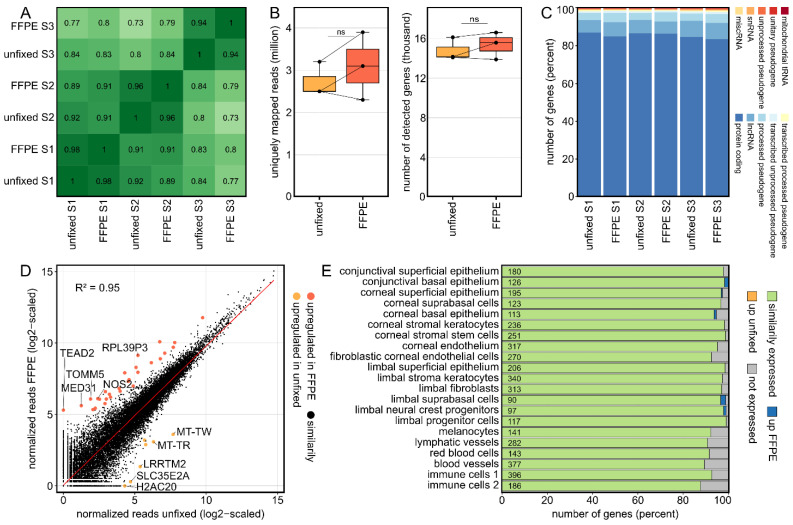
**Formalin fixation does not have a significant impact on the transcriptional profile of human corneas.** (**A**): The overall correlation coefficients between all analyzed samples revealed that the interindividual variability between the three corneas exceeded the influence of FFPE fixation, with correlation coefficients ranging from 0.94 to 0.98 between the fixed and unfixed corneal halves. FFPE: formalin fixation and paraffin embedding. (**B**): The number of uniquely mapped reads as well as the number of detected genes were comparable between fixed and unfixed corneas. The cross lines connect the respective divided sample pairs. ns: not significant. (**C**): Similarly, the proportion of different gene types such as protein coding genes or lncRNA was comparable between fixed and unfixed samples. lncRNA: long non-coding RNA; miscRNA: miscellaneous RNA; snRNA: small nuclear RNA; tRNA: transfer RNA. (**D**): Readplot visualizing differentially expressed genes (DEG) between fixed and unfixed samples. In total, there were 39,585 transcripts (99.91%) with similar expression between the two groups, with only 28 (0.07%) and 7 (0.02%) genes being up- and downregulated by fixation, respectively. The overall squared correlation coefficient (R2) between fixed and unfixed samples was 0.95. The red line corresponds to the regression line. The top five DEG per group are labeled. (**E**): Impact of FFPE processing on the expression profile of corneal cell type-specific marker genes, as previously determined by single-cell RNA sequencing. The number of marker genes for each cell type is shown within the plot.

Three cultured human corneas were divided and one half was embedded in FFPE and the other half processed without fixation. As the Pearson correlation coefficients between samples indicate, the effect of FFPE fixation was significantly smaller than the interindividual variability (Figure 1A). The number of raw reads (unfixed: 4.2 ± 0.5, FFPE: 4.3 ± 0.9; mean ± sd in millions), the mapping rates (unfixed: 2.7 ± 0.4, FFPE: 3.1 ± 0.8; mean ± sd in millions), which correspond to the proportion of reads that are uniquely mapped to a single site in the reference genome, as well as the number of detectable genes (unfixed: 14,797 ± 1152.1, FFPE: 15,360 ± 1371.9) were comparable between FFPE-fixed and unfixed samples (Figure 1B). Furthermore, the relative proportions of different gene types were comparable between both groups, with the majority of genes being protein coding (Figure 1C). Analyzing differentially expressed genes (DEG) (criteria: log2FC > 2 or <−2 and adjusted *p* value < 0.05) revealed that only 28 genes (0.07%) were upregulated in FFPE and 7 genes (0.02%) were upregulated in unfixed samples with 39,585 transcripts (99.91%) being similarly expressed (Figure 1D). The overall correlation of gene expression between FFPE-processed and unfixed samples was very high, as indicated by a squared Pearson correlation coefficient of 0.95 (Figure 1D). For a detailed analysis regarding individual cell types in the human cornea, the expression of corneal cell type-specific marker genes derived from recently published single-cell RNA-seq data [3] were analyzed (Figure 1E). The majority (91.8%) of marker genes exhibited comparable expression levels between fixed and unfixed corneas, whereas 8.1% were not detected in both groups and only 0.1 and 0.0% of markers were up- or downregulated in FFPE-processed corneal tissue. These results indicate that FFPE processing did not reveal any significant influences on the expression profile of corneal cell type-specific marker genes. 

Strikingly, 91.8% of marker genes previously detected by single-cell RNA-seq in unfixed samples were also detected in fixed corneal samples using bulk RNA-seq. This highlights the value of bulk sequencing of archived corneal samples because it enables to analyze changes in expression levels of hundreds of cell type marker genes, thus providing indirect insights into relative changes of individual corneal cell types. This is particularly relevant since single-cell RNA-seq is often not feasible on archived samples. 

### 2.2. Corneal Tissue Culture Significantly Modulates the Transcriptional Profile of Human Corneas

**Figure 2:**
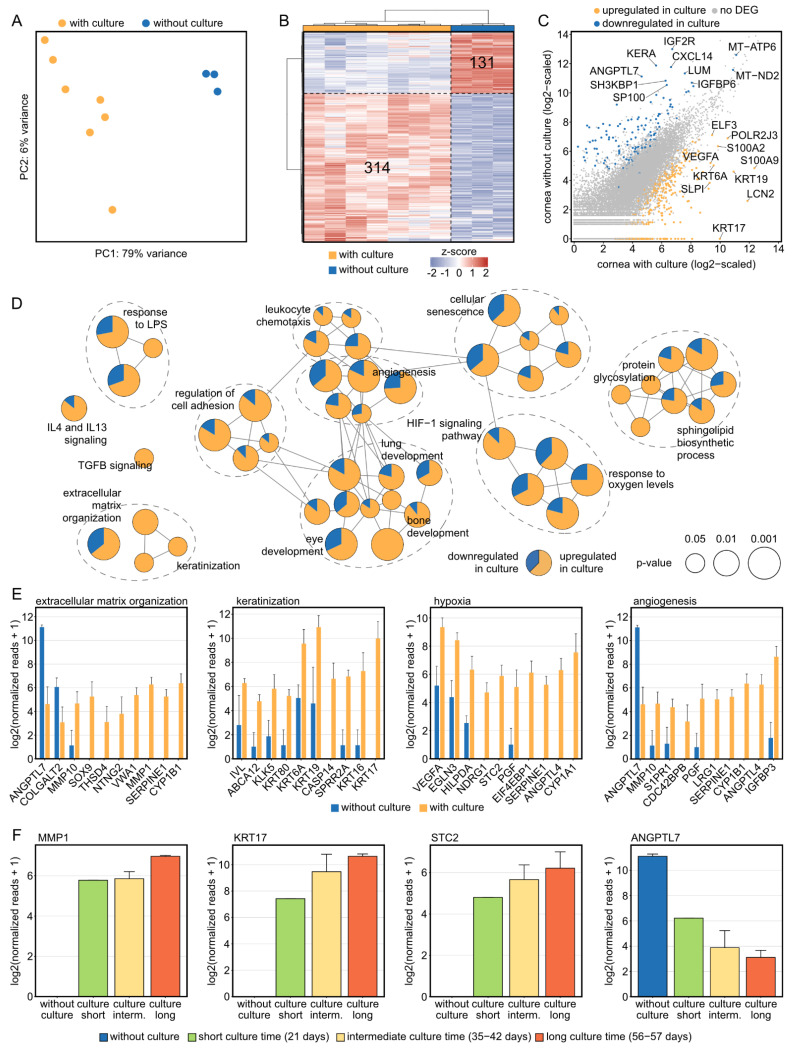
**Corneal tissue culture significantly modulates the transcriptional profile of human corneas.** (**A**) The substantial effect of organ culture is illustrated by unsupervised cluster analysis using principal component analysis. PC: principal component. (**B**): Heatmap visualizing the 314 significantly up- and 131 significantly downregulated genes by tissue culture. The z-score represents a gene’s expression in relation to its mean expression by standard deviation units (red: upregulation, blue: downregulation). (**C**): Readplot visualizing differentially expressed genes (DEG) between corneas with and without culture. The top 10 DEG per group are labeled. (**D**): Functionally grouped network analysis of enriched Gene Ontology biological processes, reactome, KEGG, and Wiki pathways in which the DEG were involved in. Enriched terms are visualized as nodes being linked based on their kappa score, which indicates the similarity of the genes linked to them. The node size represents the term enrichment significance (see legend). Representative terms of each group are labeled. The pie charts visualize the percentage of up- and downregulated genes. (**E**) The top ten DEG, which were selected based on the absolute value of the log2 fold change, are visualized for each subnetwork from (**D**). The height of the bar corresponds to mean expression and the error bar represents standard deviation. (**F**) Changes of expression in dependence of time in organ culture for genes of each subnetwork from (**E**). Number of samples per group: without culture, 3; short culture time, 1; intermediate culture time, 4; long culture time, 2.

To assess an influence of organ culture on corneal tissue, cultured human corneas from our cornea bank were compared with fixed healthy corneal tissue not stored in culture medium. Principal component analysis (PCA) indicated distinct effects of organ culture on the transcriptional profile of corneal tissue (79% of the variance in the data is explained by PC1 on the *x* axis, Figure 2A). Both the heatmap (Figure 2B) as well as the representation of DEG in a readplot (Figure 2C) demonstrated the impact of organ culture on human corneal tissue: 314 DEG were upregulated and 131 DEG were downregulated by culture (the top 10 DEG per group are labelled in Figure 2C). To gain new insights into culture-induced alterations of biological processes and pathways in human corneas, an enrichment analysis based on the identified DEG was performed. In addition to Gene Ontology biological processes, KEGG, Reactome and Wiki pathways were incorporated and the results were visualized in a functionally grouped network analysis, which grouped the significantly enriched terms (visualized as circles) based on the overlap of the associated DEG (visualized as connections between the circles). The proportion of up- and downregulated DEG for each term are shown in yellow or blue, respectively (Figure 2D). The analysis revealed that there were nine larger groups of processes and pathways which are modulated by corneal organ culture, among them extracellular matrix organization (e.g., *MMP1*, *MMP10*), keratinization (e.g., *KRT17*, *KRT16*), angiogenesis (e.g., *ANGPTL4*, *LRG1*), and hypoxia (e.g., *VEGFA*, *STC2*) (Figure 2D,E). Strikingly, the expression of genes from each category was found to be associated with culture duration, among them *MMP1*, *KRT17,* and *STC2* with an increasing expression and *ANGPTL7* with a decreasing expression with longer cultivation (Figure 2F). These results reveal substantial culture-induced modulations of a plethora of molecular processes in human corneal tissue, which considerably expands our current understanding of culture effects.

### 2.3. Corneal Tissue Culture Significantly Modulates the Corneal Cellular Composition

**Figure 3:**
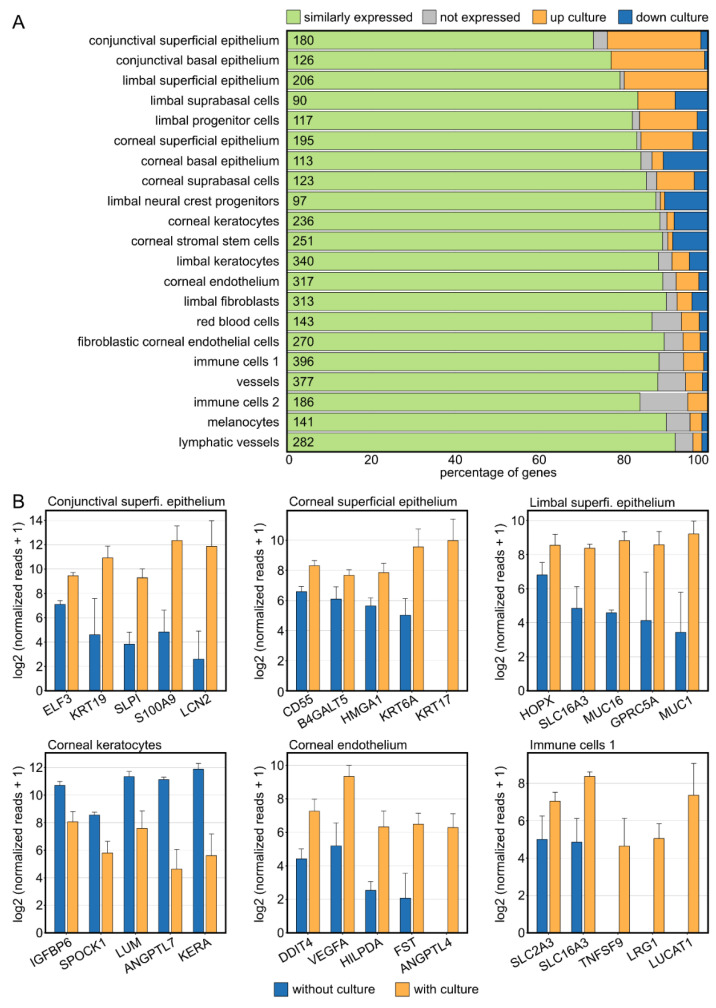
**Corneal tissue culture significantly modulates the corneal cellular composition.** (**A**): Impact of tissue culture on the expression profile of corneal cell type-specific marker genes, as previously determined by single-cell RNA sequencing. The number of marker genes for each cell type is shown within the plot. (**B**): The top five differentially expressed cell-type-specific marker genes are visualized for the most affected cell types. The height of the bar corresponds to mean expression and the error bar represents standard deviation.

In addition to culture-induced changes of biological processes and pathways, we were also interested in potential shifts in cellular composition in cultured human corneas. We therefore analyzed the expression of corneal cell type-specific marker genes in cultured corneal samples in comparison to control specimens (Figure 3A, compare Figure 1E). Strikingly, the marker of almost all cell populations exhibited culture-induced changes, with the strongest alterations seen in the epithelium (conjunctival, corneal, and limbal; predominantly upregulated by culture) and in the stroma (predominantly downregulated by culture). The top five differentially expressed genes of the highly regulated cell populations are visualized as bar graphs (Figure 3B).

### 2.4. The Corneal Immune Cell Profile Significantly Changes in Tissue Culture in a Time-Dependent Manner

**Figure 4:**
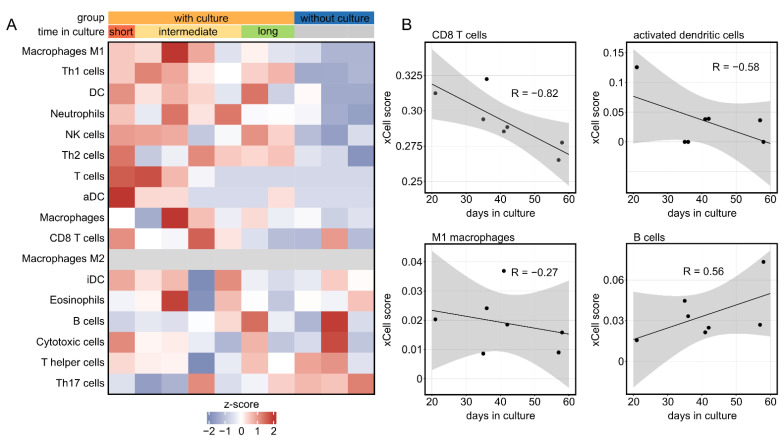
**The corneal immune cell profile significantly changes in tissue culture in a time-dependent manner.** (**A**): Immune cell profile of cultured human corneas characterized by cell-type enrichment analysis using xCell. The tool uses gene expression profiles of several immune cell types to calculate cell type enrichment scores. Each row represents one cell type, and each column represents one sample. The analysis revealed a significant influence of the culture in a time-dependent manner. Time in culture: short, 21 days; intermediate, 35–42 days; long, 56–57 days. (**B**): The influence of time in culture on the immune cell profile is illustrated by four immune cell populations.

Since the cornea harbors a high density of different immune cells, we also analyzed their profile and changes in cultured corneas. These analyses were conducted using bioinformatics cell-type enrichment analysis using xCell, which is based on well-defined gene expression signatures for different immune cell populations. xCell revealed substantial differences in the immune cell profile between cultured corneas and controls (Figure 4A). 

In addition, we observed an association between the abundance of several immune cell types and culture duration, with a trend towards decreasing cell levels with increasing culture time for most immune cell populations, including cells of the adaptive immune system, such as CD8+ T cells, as well as cells of the native immune system, such as dendritic cells and macrophages (Figure 4B). Consistent with previous findings, these results demonstrate considerable changes of the corneal immune cell profile under culture conditions, with a tendency of decreasing immune cells over time. In addition, these findings highlight the value of bioinformatics cell-type enrichment analysis to profile the immune cell composition in fixed and archived corneal tissue samples, for which single-cell RNA-seq is often not feasible. 

## 3. Discussion

Transcriptional profiling can provide unsupervised insights into the molecular profile of cells or tissues and the technology has already improved our understanding about several ocular tissues and diseases [1,2,3]. However, little is known about the transcriptional profile of corneal disease because explanted diseased corneas are usually formalin-fixed and paraffin-embedded (FFPE) for histological examination and it is not clear whether FFPE-processed corneal specimens are suitable for transcriptome profiling. Recently, it has been demonstrated that the specialized RNA-seq technology massive analysis of c-DNA ends (MACE) is feasible with FFPE samples of human conjunctiva and that the sequencing quality is not compromised in conjunctival tissue stored for extended periods of more than 10 years [4]. However, it remains elusive whether transcriptional profiling is also amenable to archived corneal samples, whose analysis is challenged by factors such as low cell and RNA quantities. In the present study, we used paired FFPE-processed and unfixed human donor corneas and applied the specialized 3′-RNA-seq technology MACE which sequences only the 3′ end of the RNA, accounting for FFPE-induced RNA degradation which mainly occurs at the 5′ end of the RNA. Additionally, each RNA molecule is barcoded with a unique molecular identifier before PCR amplification, thus reducing the PCR bias and allowing accurate quantification of gene expression [4]. Our results indicate that FFPE processing does not significantly affect several aspects of the transcriptional profile of human corneal specimens. The technology may also be valuable for the analysis of very rare entities for which a prospective sample acquisition is often not feasible. In addition, 91.8% of corneal cell type-specific marker genes previously identified by single-cell RNA-seq in unfixed samples [3] could also be detected in FFPE corneal samples using our bulk RNA-seq approach. This highlights the value of 3′ MACE bulk RNA-seq to study archived FFPE corneal samples because it enables to detect expression levels of hundreds of cell-type marker genes, thus providing indirect conclusions about relative changes of individual corneal cell types. This is of relevance since single cell RNA-seq is often not feasible on archived samples. We acknowledge that we did not investigate the influence of storage time on the transcriptional profile of human corneal samples in this study. Regarding the fact that the sequencing quality is not significantly compromised in conjunctival tissue stored for more than 10 years [4], it seems likely that similar is true for corneal tissue. However, this question needs to be addressed in future studies.

Given the finding that the transcriptional profile of fixed corneal samples is comparable to unfixed samples provides the possibility to consider fixed healthy corneal specimens as controls to study unfixed samples such as organ-cultured donor corneas. In contrast to other solid organs, which must be immediately grafted after recovery, human donor corneas are routinely stored in culture medium in eye banks before transplantation [5,6,7]. This ensures a thorough quality control as well as detailed testing for potential infectious pathogens before transplantation. It is well known that organ culture influences donor corneas, with most of the evidence being focused on the effect of culture conditions on endothelial cell density and corneal graft survival after transplantation [8,9]. However, apart from that, it is still largely unknown which other molecular processes may be altered by corneal tissue culture. 

One of the culture-induced modulations involved extracellular matrix organization and keratinization, a finding consistent with the fact that our study suggests that epithelial cell marker genes, such as *KRT16* and *KRT17*, were particularly increased by organ culture. This is in line with the morphological observation that the epithelium initially detaches in cornea bank culture and subsequently grows back as a thin, two- to three-layered epithelium. For transplantation, changes in the epithelium are of limited concern in most applications since the epithelium of the transplant is replaced by the recipient’s limbal niche assuming the latter is functional. Interestingly, *KRT6A* was also strongly upregulated under culture conditions. The gene is known to be overexpressed in conjunctival squamous cell carcinoma [2] and has been shown to promote cancer cell proliferation and metastasis in lung adenocarcinoma via epithelial-mesenchymal transition (EMT) [11]. These data imply that besides pure regeneration of the epithelium, processes such as EMT may also be activated by the culture, which is consistent with results observed in animal models under prolonged culture time [12]. In addition, our results revealed that matrix metalloproteinases (MMP), including *MMP1* and *MMP10,* were strongly upregulated under culture conditions. It is well known that MMPs are important for maintaining corneal integrity, transparency, and healing ability under homeostatic conditions [13]. However, at the same time, dysregulation of MMPs may contribute to the progressive weakening of the cornea, such as observed in keratoconus [13]. Considering an increase in *MMP1* expression over culture duration, these results may suggest that prolonged cultivation of human corneas may be disadvantageous, since processes such as EMT- or MMP-induced weakening of the cornea may occur. However, these questions need to be further addressed in future studies.

Hypoxia, angiogenesis, and cellular senescence were among the processes which were also strongly influenced by corneal culture. The cornea is naturally exposed to diurnal oxygen fluctuations arising from its avascularity and closed eyelids at night. However, extensive and prolonged hypoxia conditions, such as under prolonged organ culture, modify the composition of the extracellular matrix and the biomechanical properties of the cornea [14]. Stanniocalcin 2 (*STC2*) was one of the hypoxia-associated genes which was strongly upregulated in cultured human corneas in a time dependent manner, without any detectable expression in control samples. Recently, *STC2* was shown to be induced in the murine retina under both acute and chronic hypoxia in a HIF1A-dependent manner [15]. Interestingly, *STC2* promotes hypoxia-stimulated EMT, while silencing *STC2* reversed this process [16]. These findings imply that *STC2* may serve as a potential target to modulate culture-induced changes in human donor corneas. Organ culture also affected angiogenesis-associated factors, such as an upregulation of Angiopoietin-Like 4 (*ANGPTL4*) and a time-depended downregulation of Angiopoietin-Like 7 (*ANGPTL7*). *ANGPTL4* is involved in hypoxia-induced angiogenesis [17] and has been shown to enhance angiogenesis [18]. In contrast, the cornea-specific gene *ANGPTL7* has anti-angiogenic properties with a role in corneal avascularity [19]. In addition, the proangiogenic factor Leucine-Rich Alpha-2-Glycoprotein 1 (*LRG1*) was significantly upregulated under culture conditions [20]. These results indicate that the complex mechanisms which preserve corneal avascularity may be compromised under culture conditions. A third group of genes which were affected by the culture were genes involved in cellular senescence. Cyclin Dependent Kinase Inhibitor 2A (*CDKN2A*) was only expressed in cultured corneas without any expression in control samples. The factor represents a well-known senescence marker and an increasing percentage of cells expressed the marker in old compared to young organ donors [21]. These findings may suggest that organ culture may accelerate aging of corneal cells, although this needs to be validated in future studies. In total, these data reveal substantial culture-induced modulations of a variety of molecular processes substantially expanding the aspects of culture effects, indicating that prolonged cultivation may be disadvantageous and suggest that current culture conditions may not support optimal preservation of corneal features and may benefit from further refinement.

In accordance with previous findings, this study demonstrated that the corneal immune cell profile changes over culture time [8,9], with decreasing numbers over time for almost all immune cell populations. It is well known that corneal grafts which were in culture for more than 21 days showed less subsequent rejection than grafts stored for shorter times [8], a finding which is explained by decreasing numbers of competent immune cells in the cornea with increasing time in culture [9]. Adding modulatory factors to the culture medium that affect the differentiation status of immune cells in the graft have been demonstrated to reduce the risk of rejection [10]. Surprisingly, our data indicated that B cells exhibited an increase over time in culture. This could be explained by the fact that B cells differentiate from immature progenitor cells or that there is either no change or a slower decrease in B cells compared to the other immune cell types, resulting in a relative increase in B cells over time. It should be noted that xCell only provides relative values for the enrichment of each immune cell type. Furthermore, it is noticeable that control corneas without exposure to culture medium had lower immune cell scores compared to cultured corneas. This might be explained by relative changes of the cellular composition, with more epithelial cells in control corneas resulting in relatively lower immune cell scores. Another explanation might be that the peripheral cornea, the limbus, and the ciliary body contain significantly more immunocompetent cells than the central cornea. Thus, it remains to be considered that under culture conditions, immune cells from the periphery migrate towards the center and corneal grafts from culture therefore contain more immune cells in the central zone than native corneas. Since the control corneas were immediately fixed after procurement, this may reflect the cell-poor “as-is” condition of a normal central cornea. 

Another challenging issue in the field of corneal transplantation is the “artificial cornea” [22]. Here, researchers are striving to create biological material with physical and biomechanical properties similar to the human cornea [23] in order to ultimately circumvent the shortage of human donor tissue. Our RNA-seq approach provides a high-resolution molecular map of the human cornea, which may be instructive for the artificial tissue development process.

## 4. Materials and Methods

### 4.1. Ethics

Ethics approval was granted by the local Ethics Committee (Research Ethics Committee of University of Freiburg; Germany; reference number: 434/20). This study adheres to the tenets of the Declaration of Helsinki. 

### 4.2. Tissue Specimens

Seven human corneas (donors 1–7; see Table 1) were received from the Lions Cornea Bank Baden-Württemberg, Freiburg, Germany, after obtaining informed consent from the next of kin. Corneas with endothelium not suitable for transplantation due to a reduced corneal endothelial cell count (endothelial cell count <2000 cells/mm^2^, but above 1500 cells/mm^2^) were used for experiments. Corneas from donors presenting any transmissible diseases were excluded from the experiments. Three of these grafts (donors 5–7) were divided in two parts to investigate the effect of formalin fixation and paraffin embedding (FFPE) on their transcriptional profile: one part was FFPE processed as described below, whereas the other part remained unfixed.

Three healthy corneal samples without any contact to culture media (donors 8–10; see Table 1) were obtained from FFPE eyes, which were enucleated at our institution due to uveal melanoma. Histological examinations were performed by at least two experienced ophthalmic pathologists. There was no evidence of tumor-induced alterations in any of the corneal specimens. The use of corneal tissue for this scientific project was subject to the ethical vote as mentioned above and after separate written informed consent by each patient. The three FFPE samples were stored in our histological archive for an average of 508.3 days (range: 324–623 days).

### 4.3. Tissue Processing 

All corneas were dissected using a 9.0 mm diameter trephine. Grafts from donors 1–4 (details see Table 1) were shredded manually with a scalpel and then transferred into ReLy Buffer (GenXPro, Frankfurt am Main, Germany). Grafts from donors 5–7 were also dissected using an 9.0 diameter trephine and then cut in half with the scalpel. Samples 5a–7a were further treated as were the samples from donors 1–4. FFPE-processing of the samples 5b–7b was performed according to a standard protocol [24]. Briefly, after bisection of the corneas the tissue was fixed in 4% formalin (phosphate-buffered, pH 7) for 12 h at room temperature, dehydrated in alcohol, and processed for paraffin embedding. From each corneal sample, fifteen FFPE sections—each 4 µm thick—were collected and stored in RNase-free tubes before RNA extraction. Corneal samples 8–10 were dissected from three enucleated eyes (due to uveal melanoma), which were FFPE fixed immediately after enucleation.

### 4.4. RNA Extraction

RNA extraction from corneal samples was performed by a commercial provider (GenXPro, Frankfurt am Main, Germany) as previously described [9]. Briefly, total RNA was isolated using the Quick-RNA FFPE Kit (Zymo Research, Irvine, CA, USA). Following a DNAse I digestion using the Baseline-ZERO kit (Epicentre, Illumina, San Diego, CA, USA), the RNA concentration was measured with the Qubit RNA HS Assay Kit on a Qubit Fluorometer (Life Technologies, Carlsbad, CA, USA). The RNA quality was determined with the RNA Pico Sensitivity Assay on a LabChip GXII Touch (PerkinElmer, Waltham, MA, USA).

### 4.5. RNA Sequencing

RNA sequencing was performed by a commercial provider (GenXPro, Frankfurt am Main, Germany) using massive analysis of cDNA ends (MACE), a 3′-RNA sequencing method, as previously described [25]. We recently demonstrated that MACE allows sequencing of FFPE samples with high accuracy [4]. Briefly, barcoded libraries comprising unique molecule identifiers were sequenced on the NextSeq 500 (Illumina, San Diego, CA, USA) with 1 × 75 bp. PCR bias was removed using unique molecular identifiers.

### 4.6. Bioinformatics 

Sequencing data (fastq-files) were uploaded to the Galaxy web platform (usegalaxy.eu) [26], as previously described [27]. Quality control was performed with FastQC (Galaxy Version 0.73, http://www.bioinformatics.babraham.ac.uk/projects/fastqc/ last access on 31 May 2022). Reads were mapped to the human reference genome (Gencode, GRCh38.p13, all regions, release 39) with RNA STAR (Galaxy Version 2.7.8a) [28] using the corresponding Gencode main annotation file. Reads mapped to the reference genome were counted with featureCounts (Galaxy Version 2.0.1) [29] using the aforementioned annotation files. The outputs of featureCounts were imported to RStudio (Version 2022.02.0+443). Gene symbols were determined based on the ENSEMBL database (release 105, download on 19 January 2022) [30]. After principle component analysis [31], normalized reads and differential gene expression were calculated using the R package DESeq2 (version 1.34.0) with default parameters (Benjamini–Hochberg adjusted *p*-values) [31]. Transcripts with log2 fold change (log2FC) > 2 or <−2 and adjusted *p*-value < 0.05 were considered as differentially expressed genes (DEG). Heatmaps were created with the R package ComplexHeatmap (version 2.10.0) [32]. Other data visualization was done using the ggplot2 package (version 3.3.5). Cell-type-specific marker genes for corneal cell types were determined based on previously published single-cell RNA sequencing data of the human cornea [3]. Cell-type enrichment analysis was performed using xCell [33]. The tool uses the transcriptomic signatures of 64 distinct immune and stroma cell types to estimate the relative contributions of these cells to a bulk RNA transcriptome. Transcripts per million were calculated as an input for the analysis based on the output of featureCounts (assigned reads and feature length), as previously described [34]. xCell enrichment scores were compared between different groups using the Mann-Whitney U-test. Functional enrichment analysis was performed using ClueGO [35] in Cytoscape [36]. The analysis was run with the following specifications: two clusters, one for up and one for downregulated genes, and gene annotation: GO BP, KEGG, reactome pathways, Wiki pathways, with min level, 3; max level, 8; min number of genes, 7 and 4% of genes; Kappa score, 0.25.

## 5. Conclusions

This study reveals substantial organ culture-induced modulations of a variety of molecular processes in human donor corneal tissue, which substantially expands the aspects of previously studied culture effects. These insights may help to optimize culture conditions for human corneal donor tissue. In addition, this study demonstrates that formalin fixation and paraffin embedding does not have a significant impact on the transcriptional profile of human corneal samples. These findings pave the way for molecular profiling of a large number of archived healthy and diseased corneal samples stored in histological archives, which may improve our understanding of numerous corneal diseases. 

## Figures and Tables

**Table 1:** **Donor details.** Samples 1–7 originate from the eye bank; these specimens have been maintained in culture; samples 5b–7b were used to assess the effect of FFPE embedding; samples 8–10 are corneas fixed immediately after enucleation and were not stored in tissue culture medium (TC). Necrosis (necrotic endothelial cells in percent compared to live endothelial cells). M: male, F: female, NA: not applicable.

Donor	Age at Death [years]	Sex	Endothelial Cell Count [cells/mm²]	Necrosis [%]	Time between Death and Corneal Extraction [days]	Time in Culture [days]	Specifics	Tissue Culture (TC)	Corneal Graft Diameter
Corneal tissue in culture unfixed samples									
**1**	60	M	1898	20	1	42	/	M2	9.0 mm
**2**	59	M	949	10	1	36	Pseudophakic	M2	9.0 mm
**3**	87	F	1241	5	2	35	Corneal scar	M2	9.0 mm
**4**	82	M	803	50	0	21	/	M2	9.0 mm
**5a**	88	M	1752	3	1	57	Pseudophakic, Arcus lipoides	M2	½ 9.0 mm
**6a**	78	M	1825	3	1	56	Arcus lipoides	M2	½ 9.0 mm
**7a**	87	M	2117	10	1	40	Arcus lipoides	M2	½ 9.0 mm
Corneal tissue in culture FFPE fixed samples									
**5b**	As in 5a								½ 9.0 mm
**6b**	As in 6a								½ 9.0 mm
**7b**	As in 7a								½ 9.0 mm
Healthy corneal samples (FFPE fixed)									
**8**	55	M	Not measured	Not measured	Fixed directly after enucleation	NA	Uveal melanoma	No TC contact	9.0 mm
**9**	81	M	Not measured	Not measured	Fixed directly after enucleation	NA	Uveal melanoma	No TC contact	9.0 mm
**10**	73	F	Not measured	Not measured	Fixed directly after enucleation	NA	Uveal melanoma	No TC contact	9.0 mm

## Data Availability

The sequencing data are available in the Gene Expression Omnibus Database (https://www.ncbi.nlm.nih.gov/geo/query/acc.cgi?acc=GSE214853) (accessed on 31 May 2022)).
